# Experimental and Computational Approach Investigating Burst Fracture Augmentation Using PMMA and Calcium Phosphate Cements

**DOI:** 10.1007/s10439-013-0959-3

**Published:** 2014-01-07

**Authors:** Sami M. Tarsuslugil, Rochelle M. O’Hara, Nicholas J. Dunne, Fraser J. Buchanan, John F. Orr, David C. Barton, Ruth K. Wilcox

**Affiliations:** 1 School of Mechanical Engineering, University of Leeds, Leeds, LS2 9JT UK; 2 School of Mechanical and Aerospace Engineering, Queen’s University, Belfast, BT9 5AH UK

**Keywords:** Spine, Biomechanics, Vertebroplasty, Computational, Finite element, Calcium phosphate, Burst fracture, Trauma

## Abstract

The aim of the study was to use a computational and experimental approach to evaluate, compare and predict the ability of calcium phosphate (CaP) and poly (methyl methacrylate) (PMMA) augmentation cements to restore mechanical stability to traumatically fractured vertebrae, following a vertebroplasty procedure. Traumatic fractures (*n* = 17) were generated in a series of porcine vertebrae using a drop-weight method. The fractured vertebrae were imaged using μCT and tested under axial compression. Twelve of the fractured vertebrae were randomly selected to undergo a vertebroplasty procedure using either a PMMA (*n* = 6) or a CaP cement variation (*n* = 6). The specimens were imaged using μCT and re-tested. Finite element models of the fractured and augmented vertebrae were generated from the μCT data and used to compare the effect of fracture void fill with augmented specimen stiffness. Significant increases (*p* < 0.05) in failure load were found for both of the augmented specimen groups compared to the fractured group. The experimental and computational results indicated that neither the CaP cement nor PMMA cement could completely restore the vertebral mechanical behavior to the intact level. The effectiveness of the procedure appeared to be more influenced by the volume of fracture filled rather than by the mechanical properties of the cement itself.

## Introduction

The annual incidence of high-energy spinal fractures is reported to be as high as 150,000 in the United States alone.^49^ Traumatic burst type fractures often occur when the spine is loaded under high-rate axial compression, frequently coupled with flexion or extension.^24^,^35^,^63^ The most common causes of such fractures are falls from height, motor vehicle accidents or sports injuries^3^,^28^ and prevalence in the younger population (20–40 years of age) is widely reported.^3^,^7^,^28^ The term “burst fracture” was first defined by Holdsworth^21^ in 1963 as a stable fracture brought about by high impact axial compression. It was deemed to be stable due to the location of the injury being anterior of the posterior ligament complex. Denis^13^ later suggested instability could not be determined by the compromise of the posterior ligament complex alone and proposed that the rupture of the posterior longitudinal ligament and the annulus fibrosus also implies instability. The classification of the injury and assessment of the stability are critical if appropriate treatment is to be administered, however this is not trivial because the concept of spinal stability is ambiguous and often difficult to define.^45^ In addition, the injuries sustained following spinal trauma can vary significantly in severity^35^ often causing a wide variety of symptoms and fracture patterns.

Surgical intervention for traumatic burst fractures is often highly invasive, especially when instrumentation, either posterior and/or anterior, is involved^60^ and as such the decision on whether to treat surgically is strongly debated. Vertebroplasty, involving the percutaneous injection of liquid bone cement into the fracture site, has only recently been considered as a possible alternative to traditional surgical treatment of burst fractures, and there is still only a limited number of clinical case studies reporting its use.^1^,^9^,^10^,^15^,^26^ These studies concluded that the treatment has the potential to reduce reliance on painkillers and increase mobility.^26^

Vertebroplasty benefits from being a minimally invasive procedure; however the poly (methyl methacrylate) (PMMA) cement commonly used may not be optimal for traumatic burst fracture augmentation. It is not osteoconductive, so it cannot be fully integrated with bone. In addition, the cement experiences a high exothermic temperature during polymerization reaction, which has been shown to exceed 70 °C,^14^,^16^,^29^,^46^ potentially leading to surrounding tissue necrosis.^19^ Injectable calcium phosphate (CaP) cements have been considered as an alternative to PMMA as they have the potential to be osteoconductive, encouraging bone in-growth and remodeling. Additionally, the setting reaction occurs at body temperature. However, CaP cements are often associated with long setting times and are characteristically brittle. A limited number of studies exist that have investigated CaP cement for the augmentation of burst fractures,^34^,^36^,^52^ and one clinical case study has reported successful application and favorable outcomes following augmentation of an osteoporotic burst fracture using a CaP cement variation.^37^

The concept of using finite element (FE) analysis for modeling the spine is relatively new, but in recent years an increasing number of studies have adopted the technique to evaluate specific aspects of the complex biomechanics of the spine.^30^,^38^,^50^,^65^,^66^ In addition, there are examples of the application of FE modeling for the assessment of the vertebroplasty procedure on osteoporotic vertebrae using PMMA cement.^2^,^11^,^25^,^47^,^53^,^59^,^61^,^62^ The aim of many of these studies was to investigate the consequences and long term complications observed following vertebroplasty, such as adjacent level vertebral fracture.^58^ Due to the complicated interactions between the bone cement and the cancellous bone, which is often mechanically compromised, the predictions of such models have been associated with poor agreement with the results of concurrent experimental testing, as concluded by Wijayathunga *et al.* 61 There have been a number of experimentally validated studies that have used computational models to understand the mechanisms that occur during the burst fracture event in order to better understand how the fracture is instigated and propagates.^27^,^48^,^63^ In contrast, FE techniques do not yet appear to have been used to model burst fractured vertebrae following augmentation, or to investigate the structural performance of different augmentation cements.

In this study, it was hypothesized that a newly developed CaP cement could be used to restore the mechanical behavior of traumatically fractured vertebrae under static loading conditions, to similar values obtainable from a PMMA augmentation. The aim was to use a combination of FE modeling and experimental testing to evaluate, compare and predict the ability of the two augmentation cements to restore the mechanical stability. Since the definition of spinal stability is somewhat ambiguous, standardized engineering measurements of stiffness and strength were used to compare treated and non-treated fractured specimens against intact vertebrae. The FE models were then utilized to determine the effect of cement modulus on the stiffness of the specimens.

## Methods

### Outline

**Figure 1:**
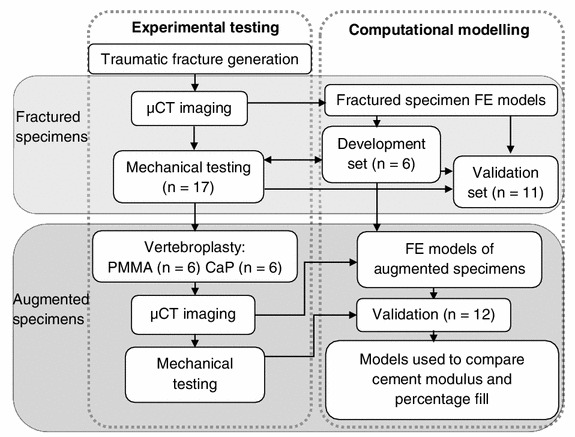
Experimental and computational methodology used within this study

An outline of the experimental and computational methods is presented in Fig. 1, and described in further detail below.

### Experimental Tests

**Figure 2:**
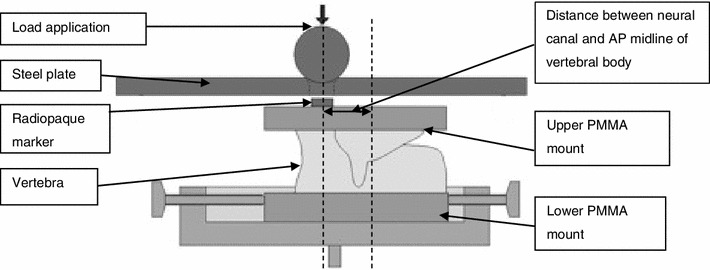
Loading Scenario and radiopaque marker location^51^

An experimental procedure for fracture generation and augmentation of porcine vertebrae was developed previously and is described in detail elsewhere.^52^ In brief, traumatic fractures were generated in a series of porcine vertebrae using a drop-weight method. The fractured vertebrae were potted in PMMA end caps and imaged using micro computed tomography (μCT) with a voxel size of 74 *μ*m (μCT80 Scanco, Switzerland). The fractures generated within this study were predominantly Denis type-A fractures. This was determined using a scoring technique adapted from Panjabi *et al.*,^44^ whereby a grid is superimposed on to the images of the fractured vertebral body and the vertebral sections within the grid squares are assigned a score based on the severity of the fracture between 0 (no fracture evident) and 2 (severe or multiple fractures). This was normalized for a selection of images spanning the height of the damaged vertebrae and compared to a threshold score, which determined the specimen’s inclusion in the study. This technique is described in more detail in previous studies.^51^,^52^ The selected specimens (*n* = 17) were then tested under axial compression up to a certain predefined axial load in a materials testing machine (Instron 3366 10kN, Instron, USA) at a loading rate of 1 mm/min. The load was applied *via* a ball within a steel housing to allow the upper cement end cap to tilt (Fig. 2). The stiffness, which was used to compare to predictions obtained from FE models, was determined as the largest gradient of the load–displacement curve obtained over a 0.6 mm displacement range, based on previous studies.^61^ A radiopaque marker embedded within the top cement end cap was used to locate the specimen relative to the loading point (Fig. 2).

Twelve fractured vertebrae were selected at random and each specimen underwent a bi-pedicular vertebroplasty procedure using either a laboratory grade PMMA cement (WHW Plastics, Hull, UK) (*n* = 6) or a CaP cement variation (*n* = 6) designed and developed at Queen’s University, Belfast.^39^ The formulation of the CaP cement was similar to that used in the previous study,^52^ however a higher liquid to powder ratio of 0.5 mL/g was used to provide a less viscous, more injectable material, but with a lower elastic modulus.^40^

### Development of Models of Fractured Vertebrae

FE models of the fractured vertebrae were first generated from the μCT image data. Each specimen was converted from DICOM into TIF format and the images were imported into an image-processing package (ScanIP version 4.2, Simpleware Ltd., UK). The image data for each specimen was down-sampled to reduce the voxel size from 0.074 mm to 1 mm, using a partial volume effect algorithm.

The models were segmented in the following way: A threshold operation was used to create a mask which captures the bone regions only, leaving the fracture gaps and mounting plates un-segmented. The bone mask was duplicated and a morphological closing operation using a kernel size of 2 pixels^3^ was implemented to fill in the region of copied bone mask that was deemed to be fracture region. A Boolean subtraction was then used to create separate non-overlapping masks for the bone and the fracture. Any regions deemed to be not part of the fracture were then removed manually from the fracture mask. The cement mounting plates and delrin markers were segmented in a similar way.

In total, five separate material regions (“masks”) were created for the vertebra, the two PMMA cement mounts, the fracture gap and the radiopaque marker. The location of the marker was recorded for the subsequent FE models and it was then removed from the model since no load was transmitted through it.

Following the threshold operations the models were meshed, using a mix of tetrahedral and hexahedral elements, with an approximate element size of 1 mm^3^ (by direct voxel to element conversion) which has been shown previously to be sufficient to predict the stiffness of vertebrae modeled using the same approach.^23^ During the image to mesh conversion, a smoothing operation was used based on the underlying grayscale of the image, this ensured a closer morphological match to the underlying image was achieved (Scan FE version 4.2, Simpleware Ltd., UK).

Material properties were then assigned to the model. All materials were assumed to be linearly elastic and isotropic. The PMMA cement end caps were assigned an elastic modulus of 2.45 GPa^61^ and a Poisson’s ratio of 0.3.^18^,^42^,^61^ The elastic modulus of each vertebral bone element was linearly related to the gray-scale of the corresponding image voxel, since previous studies have shown that the use of a linear relationship between gray-scale and elastic modulus yielded similar accuracy to the use of power–law relationships.^61^ A Poisson’s ratio of 0.3 was used for all the bone elements; it has previously been shown^23^ that the model predictions were relatively insensitive to the choice of this parameter.

Due to the large number of fracture surfaces within the vertebrae, the implementation of contact between them would likely lead to mesh penetration and model convergence issues during solution. In addition, any contact interaction definition would have been based on assumptions which would not be possible to experimentally validate. Instead, the fracture gaps were filled with a relatively low modulus material (1 × 10^−9^ GPa, Poisson’s ratio, *u* = 0.3) to simulate the fracture gaps allowing relative movement of the bone fragments without the surfaces overlapping each other.

A sensitivity study was conducted in which the elastic modulus of the fracture gap was changed incrementally for one of the specimens until its effect on the vertebral stiffness was undetectable; it was then further reduced by three orders of magnitude, to ensure the fracture material did not affect the vertebral stiffness of any of the other specimens which may have a slightly more severe fracture pattern.

**Figure 3:**
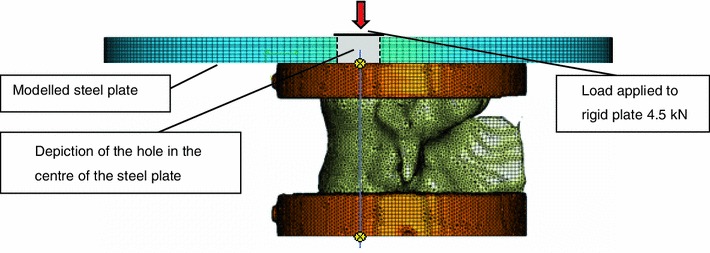
Example of FE model of single vertebra specimen

Each model was imported into a FE software package (Abaqus version 6.9, Simulia, Dassault Systemes, France). Datum axes were created using the coordinates obtained from the position of the radiopaque marker. A steel plate was added to the model (*E* = 210 GPa, *υ* = 0.3, mesh size = 1 mm^3^) and positioned on top of the specimen using the datum axes. A compressive load of 4.5 kN acting vertically downwards was applied to the model *via* an analytical rigid disk which was positioned over the steel plate, Fig. 3. The analytical plate was constrained so that it could not move in the horizontal plane, but was free to rotate; this represented the ball contact between the material testing rig and the steel plate described in the experimental testing. Boundary conditions were applied to the model on the flat base of the lower PMMA plate to replicate the experimental load conditions. The stiffness values of the specimens were calculated using the displacement of the loading point on the plate that occurred at 4.5 kN of load.

In order to determine the appropriate factor for converting image gray-scale data into the elastic modulus values for the bone, a development set of six models was initially used. The conversion factor was iteratively changed until the mean error between the experimental stiffness values and the corresponding model predicted values was minimised. To determine the accuracy of this method, the derived conversion factor was applied to the bone elements in the remaining models (*n* = 11) and their predicted stiffness values were compared to the corresponding experimental values.

### Development of Models of Augmented Specimens

A similar methodology to that described in the previous section was used to model the specimens following cement augmentation. The μCT images of the specimens were imported into the image processing software and masks representing each of the components were generated as before. An additional mask representing the cement injected into the fracture site was made using appropriate threshold operations. Based on the cement type injected, these regions were subsequently assigned properties of 1.035 and 0.585 GPa for the PMMA and CaP materials respectively, determined from experimental compression tests^39^,^51^; a Poisson’s ratio of 0.3 was applied for both cement types. For the bone material properties, the relationship between the image grayscale and the elastic modulus determined from modeling the fractured specimens was applied. After the models were solved, the predicted stiffness values were calculated by dividing the load applied to the top of the analytical rigid plate by the displacement recorded in the axial direction, these stiffness values was compared to the corresponding stiffness values recorded experimentally.

### Analysis of Cement Properties and Fracture Fill

From the μCT images of the pre- and post-augmentation specimens, the volumes of the cement and fracture void regions were determined using the image analysis software (ScanIP version 4.2, Simpleware Ltd., UK). The volume of cement was then calculated as a percentage fill of the total fracture volume for each specimen.

The FE models were then used to evaluate the relative importance of cement modulus and percentage fill of the fracture by altering the material properties of the cement.

The cement regions in all of the augmented specimens (*n* = 12) were first assigned an elastic modulus representing the PMMA cement (1.035 GPa). Then the cement regions were all assigned the modulus of CaP cement (0.585 GPa). In both cases, the models were solved and the stiffness predictions were obtained. This FE modeling method enabled comparison of two very different cement types, with a large variation in modulus and injection volumes.

### Statistical Analysis

The experimentally obtained mean stiffness and ultimate failure strength of specimens augmented using the CaP and PMMA cements were compared with the values obtained from the independent set of fractured specimens (*n* = 5) that were not augmented with either cement. Using results obtained previously from a study of intact porcine vertebrae,^52^ the stiffness and failure load values were also compared against the values obtained for the fractured and two augmented groups of specimens using a one-way analysis of variance (ANOVA, *α* = 0.05, the null hypothesis was that the means of the groups were equal) and* post hoc* tests [Tukey–Kramer (T–K) test, *α* = 0.05, for stiffness and failure load]. A two-tailed, unpaired *t* test was used to compare the percentage fracture fill of the two cements injected (*α* = 0.05), the null hypothesis was that the mean values of the percentage fracture fill resulting from injection of the two cements were equal. The dependent variables were the stiffness and the failure load and the independent variable was the state of the specimen under test (intact specimen, fractured with no treatment, fractured/augmented using PMMA and fractured/augmented using CaP 0.5 l/p ratio). The agreement between the experimental results and those predicted from the FE models was assessed graphically using mean-difference plots as proposed by Bland and Altman.^4^ In addition, the agreement was assessed using the concordance correlation coefficient described by Lin^32^ which measures the variation from the line of perfect fit (i.e., the 45° line through the origin). The Pearson’s correlation coefficient between percentage of the fracture filled and the increase in specimen stiffness following vertebroplasty was also calculated for the different cements (two-tailed test, *n* = 12, *α* = 0.1).

## Results

### Experimental Results

From the analysis of the μCT images, the mean proportion of the fracture voids filled with CaP and PMMA cement was 36% (SD = 12.5%) and 53% (SD = 5.5%) respectively. This suggests that the PMMA cement penetrated the fracture gaps significantly better than the CaP cement (*p* = 0.025).

**Figure 4:**
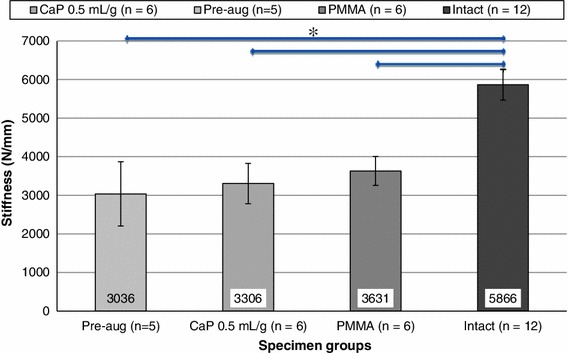
Comparison of mean of specimen stiffness (± SD) pre- and post-augmentation using the different cement types (*significant difference *p* < 0.05)

It was found that the fractured specimens had a significantly lower mean stiffness than the intact specimen group (ANOVA: *F*(3,44) = 22.9, *p* = 4.3 × 10^−9^, T–K test: *α* = 0.05). The mean stiffness values of the augmented sets of specimens were also found to be significantly lower than those of the intact specimens (T–K test: *α* = 0.05), indicating that neither of the cements injected were able to fully restore axial stiffness to the intact specimen level. Furthermore, neither of the cements injected were found to increase the mean specimen stiffness significantly, when compared to the fractured specimens (T–K test: *α* = 0.05), Fig. 4.

**Figure 5:**
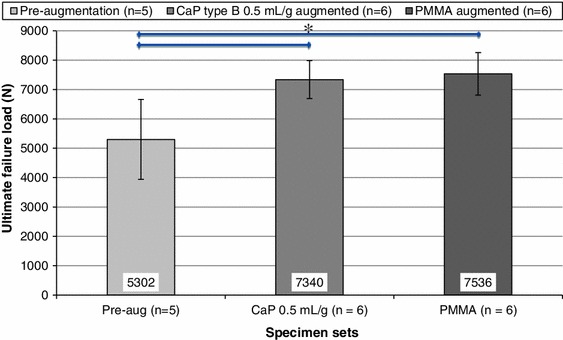
Comparison of mean of specimen ultimate failure load (± SD) pre- and post-augmentation using the different cement types (*significant difference *p* < 0.05)

Significant increases in failure load were found for the specimen sets augmented with PMMA and CaP cement when compared to their pre-augmentation values (ANOVA: *F*(2,15) = 7.16, *p* = 0.0066, T–K test: *α* = 0.05), Fig. 5, despite the modulus of the CaP cement being lower than that of the PMMA cement used (0.585 and 1.035 GPa, respectively).

### Computational Model Validation Results

**Figure 6:**
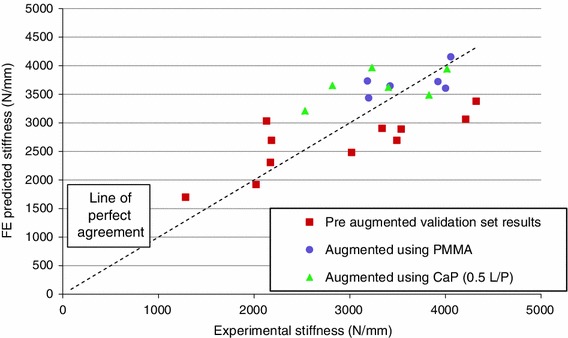
FE model stiffness predictions compared to experimental results for traumatically fractured and augmented specimens

**Figure 7:**
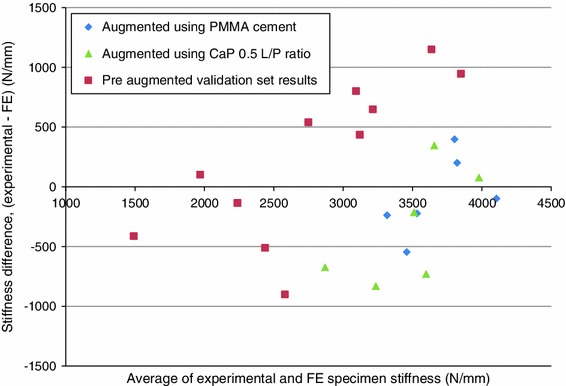
Bland–Altman difference plot of specimen stiffness pre and post augmentation, difference (experimental–FE) vs. average of values obtained from experimental and FE methods

The agreement between the experimental and FE-predicted stiffness of the traumatically fractured and augmented specimens is presented in Figs. 6 and 7. The FE models were found to predict the stiffness of the fractured and augmented specimens reasonably well based on the results presented, with a concordance correlation coefficient of 0.69^32^ excluding the development set results. The absolute mean percentage errors of the FE stiffness predictions when compared to the experimental results for the fractured and augmented sets were 20.9 and 12.1% respectively.

### Computational Analysis of Cement Properties and Fracture Fill

From the results comparing the predicted stiffness of the models following augmentation, it appears that the FE models were more sensitive to the percentage of the fracture that was filled with cement than to the cement modulus. There was a moderate positive correlation between percentage of the fracture filled and the increase in specimen stiffness following vertebroplasty, especially for the PMMA augmented specimens Pearson’s correlation coefficient = 0.51 (*p* < 0.10) for PMMA and 0.48 (*p* > 0.10) for CaP.

## Discussion

The use of vertebroplasty for the treatment of osteoporotic fractures has received considerably more attention^22^,^31^,^56^,^57^,^64^ than its application in traumatic spinal fracture management.^34^,^36^,^52^ There have also been considerable efforts to improve the mechanical properties and the delivery of CaP cements^5^,^6^,^20^,^39^ in an attempt to develop a superior, osteoconductive alternative to PMMA, for use in vertebroplasty. In contrast, few groups have used FE modeling to investigate the effects of vertebroplasty on the biomechanics of the traumatically injured spine. The aim of the present study was to use a combined biomechanical testing and computational approach to evaluate, predict and compare the ability of PMMA and CaP cement to restore stiffness and ultimate failure load of traumatically fractured vertebrae following vertebroplasty.

### Discussion of Methods

The use of specimen-specific FE models enabled direct comparisons to be made between the model predictions and the corresponding experimental results. The models were found to predict the stiffness of both the fractured and augmented specimens effectively when compared to the equivalent experimental results, especially when considering the large spread found in the experimental stiffness data (1284–4322 N/mm). In this study, traumatic fractures were generated that yielded large regions of fractured material and gaps within the specimen. The approach used to represent both the fracture gaps and the augmented region appear to have been more successful than similar methods used to simulate augmentation of compression fractures in osteoporotic vertebrae, where large errors in the predictions have been found.^61^ The good levels of agreement found here provided confidence that the traumatic fracture FE models could be used to investigate different augmentation scenarios; it would not be possible to examine these different scenarios in a controlled way experimentally. The specimen-specific approach also meant that a range of different fracture severities were represented in the study rather than a single “standardized” fracture.

One of the most important steps in generating the models is the down-sampling of the image data. A previous study where the factors influencing FE vertebral model predictions were investigated,^23^ found a voxel size of 2 mm was optimum for modeling single vertebral bodies since larger sources of error arose from inaccurate location of applied boundary conditions than from a reduction in element size below this value. A voxel size of 1 mm^3^ was chosen in the present study (resulting in approximate element size of 1 mm^3^) to allow for the inclusion of the more complicated geometry of the posterior elements and fracture gaps within the vertebra. A reduction of voxel size smaller than 1 mm^3^ would mean that the voxel size would begin to be within the size range for the trabecular spaces within the bone (0.35–0.77 mm).^54^,^55^ Other studies^67^ have also found that there was some evidence of convergence at larger element sizes (where the element sizes were much larger than the trabecular spaces), but as the image resolution neared that of the trabecular structure itself, there was instability, since the elements were beginning to represent either trabecular space or trabecular bone, rather than the average of the two. For this reason further reductions of the voxel size may not significantly improve the model predictions for vertebral stiffness especially when compared to the increase of computational expense and extra time required to process the images.

A limitation of this study was in the use of a porcine model to represent human spinal tissue; this was discussed in detail in a previous study.^52^ In brief, a number of studies have analyzed or compared human and porcine tissue^8^,^12^,^54^,^55^ concluding that porcine vertebral bone is a reasonable model for human tissue. However, porcine vertebrae was far less porous than that of available human cadaveric tissue, which is often from a much older cohort than the patients typically susceptible to traumatic burst fracture.^3^,^7^ While it is likely that the porosity of the porcine bone limited the penetration of the cements into the trabecular structure, the model used here effectively represents the worst case scenario for the injection of the cement. When considering the causes of spinal burst fracture and the mean age of the typical patient presented, the porcine model was therefore deemed acceptable as a representation of young human tissue.

### Discussion of Results

From the experimental results, the CaP cement tested in this study increased the axial stiffness and ultimate failure load of the vertebrae to similar levels when compared to the PMMA cement despite the differences between the compressive moduli of the two materials. The penetration of PMMA into the fracture gaps of the vertebrae was significantly better (*p* < 0.05), despite studies indicating CaP cement has a lower viscosity than PMMA cement prior to injection, measured using a parallel plate rheometer.^17^,^41^ The difference in injectability, is therefore thought to be due to “filter pressing” occurring during injection of the CaP cement, this is defined as the separation of the liquid from the cement suspension, occurring when the pressure necessary for cement extrusion is greater than the pressure required to filter the liquid through the cement powder.^6^ Following the injection of the CaP cement, there was evidence of some filter pressing having occurred from examination of the contents of the syringe barrel, however the extent of this was less when compared to the more viscous CaP cement (L:P 0.35 mL/g) used in the previous study.^52^ This is evidenced by the difference in the mean fracture void fill recorded, CaP L:P 0.5 mL/g cement 36% (SD ± 12.5%) void fill compared to 27% (SD ± 13) void fill for CaP L:P 0.35 mL/g.^52^ While these differences were not statistically significant, the less viscous cement would seem to improve the injection performance, a similar finding was also concluded in a study by Dunne *et al.*,^17^ in which, increasing the liquid to powder ratio of the CaP formulation from 0.35 to 0.5 mL/g made the cement more workable, improved the mixing and considerably increased the injectability, 61% compared to 95% respectively.^17^ These findings indicate that the viscosity of the cement and resulting penetration of the cement into the fracture volume appears to have a prominent influence on the restoration of the mechanical stability of the vertebra.

When comparing the vertebral stiffness of the augmented specimen sets, it is clear that, while there is some improvement of the stiffness (p > 0.05) over the pre-augmentation values (Fig. 3), it is still significantly lower (*p* < 0.05) than the stiffness of the intact specimens.^52^ In contrast, the ultimate failure load of the augmented specimens, for both cement types used, was significantly higher than the pre-augmented case (*p* < 0.05). Due to limitations of the load cell used (10kN maximum) the ultimate failure load could not be fully determined for the intact specimens. This however does seem to suggest that the augmentation procedure did not fully restore ultimate failure load to intact levels. These are important findings when considering the use of PMMA as the augmentation material since PMMA is not osteoconductive and has no capacity to be resorbed and replaced by bone. It is therefore reasonable to assume that the stiffness and strength increases brought about by the PMMA augmentation are likely to be at their maximum immediately after the cement has set. In contrast, when injected *in vivo*, the CaP cement is capable of being resorbed and facilitates the occurrence of fracture healing and bone regrowth,^43^ thereby encouraging longer term stability of the vertebrae. Therefore considering the similarity in the performance of both of the cements tested in the present study, the CaP cement may be the preferred option in these cases.

The results obtained from the FE models to assess the effect of cement modulus and percentage fracture fill demonstrate greater increases in stiffness resulted as a consequence of volume of cement injected rather than by the difference in modulus between the cements tested. This substantiates the experimental findings, suggesting that while the cement modulus is an important factor in the restoration of vertebral stability, the injectability and penetration of the cement into the fracture site also has a very prominent effect. The CaP cement used in the present study appears to perform similarly when compared to the PMMA cement, and benefits such as bioactivity and the possibility of bone remodeling may further improve the cements performance in the longer term. However, the handling and delivery of the CaP cement requires further improvement and standardisation in order to enhance the predictability of the cement type in situ and for the cement to be a viable alternative to PMMA cement. This has been the focus of a number of studies in recent years.^6^,^20^

While this study presented a static loading model, further work is required to examine the performance of CaP cements under cyclic loading conditions. It is particularly relevant to study the fatigue behavior of the characteristically brittle, CaP cements, as any failure that occurs in this case is likely to cause an immediate reduction in structural integrity of the cement, potentially reducing the stability of the fracture. Failure in such a manner may cause the cement to fragment and particles subsequently, may trigger osteolysis in the augmented vertebra, this is an issue that would require further investigation following appropriate fatigue testing. With consideration to this issue, other authors have looked into combining PMMA and CaP cements together to obtain the benefits of both the materials, in particular the desired fatigue and failure response of PMMA and the potentially osteoconductive properties of CaP, thus improving the bioactivity of the PMMA cement.^33^

This study presented a combined computational and experimental approach to investigate the biomechanical performance of two cements for the augmentation of traumatically-fractured vertebrae. The results indicated that neither the CaP cement nor PMMA cement could completely restore the vertebral behavior to the intact level, and that the effectiveness of the procedure was more influenced by the amount of fracture void filled with cement than by the mechanical properties of the cement itself. This indicates that a low modulus CaP cement variation may be effective if it can be injected to penetrate all of the fracture voids. The methods presented here will be used in future to investigate other clinical scenarios and augmentation options.
